# Corporations and Health: The Need to Combine Forces to Improve Population Health

**DOI:** 10.34172/ijhpm.2022.6687

**Published:** 2022-05-18

**Authors:** Mélissa Mialon, Gary Fooks, Katherine Cullerton, Clara Gómez-Donoso, Hernando Salcedo Fidalgo, Rima Nakkash, Jennifer Lacy-Nichols

**Affiliations:** ^1^Trinity College Dublin, Dublin 2, Ireland.; ^2^Aston University, Birmingham, UK.; ^3^University of Queensland, Brisbane, QLD, Australia.; ^4^University of Navarra, Pamplona, Spain.; ^5^FIAN Colombia, Bogota, Colombia.; ^6^Health Promotion and Community Health Department, Faculty of Health Sciences, American University of Beirut, Beirut, Lebanon.; ^7^Global and Community Health Department, College of Health and Human Services, George Mason University, Fairfax, VA, USA.; ^8^University of Melbourne, Melbourne, VIC, Australia.

**Keywords:** Commercial Determinants of Health, Ethics, Conflicts of Interest

## Abstract

The recent concerns raised about commercial determinants of health (CDoH) are not new. Numerous organizations around the world are working on these issues. These groups have emerged in response to specific issues and contexts and bring with them a diversity of interests, worldviews and strategies for change. In creating the ‘Governance, Ethics and Conflicts of Interest in Public Health’ network in 2018, our hope was to broaden our engagement with other actors advocating for change and strengthen our collective efforts. For academics, this requires moving further beyond the collective comfort zone of peer-reviewed publications, working with the media and those with political expertise, and learning from and supporting other stakeholders with a common vision.

 People are living longer, but also experiencing more years of disability and ill-health from a growing range of chronic conditions.^1^ In addition, human activity is having destructive environmental impacts, which – if not addressed – will have devastating, long term effects on human health.^2^ At the center of these issues lie corporations, particularly global companies, to the extent that the term ‘commercial determinants of health’ (CDoH) has been recently coined to describe the corporate products and practices that drive ill-health globally.^3^ CDoH encompasses^3^: (*i*) the production of ‘unhealthy commodities’ (such as tobacco, alcohol, ultra-processed food products, and fossil fuels); (ii) harmful corporate practices, such as bad working conditions or aggressive marketing; (*iii*) political practices, such as lobbying, to secure favorable policy environments for the consumption of unhealthy commodities; and (*iv*) more deep-seated drivers of ill-health, such as neoliberal economic policies – which exacerbate social and, therefore, health inequities.

 We are already observing responses to these harmful corporate practices. CDoH are for example attracting attention of global health agencies like the World Health Organization (WHO).^4,5^ Governments have made efforts to restrict certain corporate activities and impose appropriate penalties, particularly in the case of the tobacco industry.^6^ With this in mind, we formed the ‘Governance, Ethics and Conflicts of Interest in Public Health’ network, in 2018.^7^ Our mission is to share and facilitate research, knowledge exchange, and policy dialogue on governance, ethics and conflicts of interest (COI) in public health, issues that align closely with the CDoH.^7^ Our network is driven primarily by academics, but scholars do not necessarily have the resources and skills to make their voices heard beyond academia. Moreover, CDoH are not a core part of research funders’ agenda.^8^ The result is a lack of academic capacity relative to other areas to support our work.^8^

 We are arguing that collaboration amongst a wide range of stakeholders will certainly be key to address the CDoH.^9^ Greater professional diversity in networks such as ours – which brings together actors with different skill-sets and capacities – will not only enhance understanding of the nature and risks associated with the CDoH, but also strengthen the hand of groups with an interest in how these take effect, and encourage action at local and national levels. Progressing our goals requires persistent engagement across this spectrum of individuals and their institutions.^8^ In addition, collaboration could strengthen research translation and advocacy around CDoH.^8^

 In particular, group advocacy and activism have the potential to mitigate negative health impacts and promote health. Strategies may include product boycotts, shareholder activism, public protest, awareness-raising, legal action and lobbying governments to restrict corporate activities and/or impose appropriate penalties.^9^ We can learn from past successful advocacy efforts from civil society organizations and social movements, which have proven key to influencing public policy and public discourse on corporations and health.^10,11^ One of those example is the International Baby Foods Action Network, together with other civil society organizations, which successfully led action against the tragic marketing practices of infant formula in Africa and Asia that resulted in the deaths of millions of babies in the 1970s.^12^ Public health movements have also been successful in curbing tobacco smoking and calling out the harmful practices of the tobacco industry.^10^ For instance, in Russia, the formation of the Anti-Tobacco Advocacy Coalition, by pointing out industry efforts to undermine tobacco control, changed the public’s view of the tobacco industry, which in turn helped to bring about a decline in smoking rates.^11^ In Lebanon, successes in resisting tobacco industry influence and strengthening tobacco control were also only possible when academia, civil society, and the media were able to advance a tobacco control agenda together.^13^ Equally, in Latin America, coalitions of civil society organizations, working directly with affected communities, are currently advocating for legal reform aimed at promoting healthy diets and preventing diseases, with an emphasis in addressing undue influence from the food industry on public health policy.^14^

 We acknowledge that academics, journalists, the public health community, members of civil society organizations and government officials all have different approaches and views with regard to these issues. For example, a recent study by Cullerton et al^15^ found that there are stronger levels of agreement on the need to reject corporate funding amongst individuals from civil society organizations and governments than amongst researchers, particularly when funding is from the ultra-processed foods industry. Unease over undue influence and COI also meant that individuals from civil society organizations and governments were less likely than academics to enter into partnerships with corporations, even where partnership might potentially advance public health goals.^15^ Cullerton et al noted that different groups have different interpretations of the phrase “advance public health goals.”^15^ This points to a key tension in terms of how different actors interpret undue influence, COI and other risks associated with CDoH. Some actors and organizations take a more uncompromising approach to corporations, whereas others take a more nuanced view towards engagement. Indeed, the absence of a consensus definition of the CDoH highlights the ambiguities and grey areas involved in identifying, addressing, and preventing undue influence from commercial actors and COI in the first place.^8^

 Given the need to tackle the aforementioned challenges, we have developed a website to be used as a resource for those interested in addressing these issues (https://www.aub.edu.lb/fhs/Pages/GECI.aspx). We aim to provide a clearinghouse for the latest evidence and activities regarding governance, ethics and COI in public health. The website contains a list of scientific publications on CDoH. It also provides access to our newsletter and a series of webinars and short online videos on CDoH, targeted at a general audience, which can be used in capacity building by universities, civil society organizations and the media. We are also gathering a list of journalists and newspaper articles on our areas of interest and have compiled a directory of organizations, from different sectors, working on governance, ethics and COI in public health (originally published here^6^). We invite readers to share the details of any other organizations and coalitions we may have missed.

 We encourage academics, journalists, the public health community, and civil society organizations, to strengthen their links around a common set of goals aimed at protecting population health. To this end we call for:

- Academics to support local efforts, using their research and knowledge, to counter or prevent undue influence from corporations on public health policy, research and practice. This could be done in the countries where they work, but their support could also benefit other countries, particularly when there are limited resources for civil society organizations and social movements to collate and present academic knowledge to policy-makers.
- Academics, journalists, the public health community and members of civil society organizations to join efforts in building databases on COI like those created for policy-makers in France and Chile.^6^ Healthcare professionals are also encouraged to take this action with regards to payments from the pharmaceutical industry, as it has already been done in the US and some countries in Europe.^6^ These could be complemented by databases on corporations, outlining how their activities affect population health. Such databases are likely to be invaluable for awareness raising among professionals, advocates, and public officials interested in understanding, limiting and preventing CDoH.
- Civil society actors to support researchers in co-producing policy briefs on CDoH to facilitate the translation of research beneficial to the goals of civil society organizations and social movements, and to the broader public.

The concerns we raise about CDoH are not new. Numerous organizations around the world are working on these issues. These groups have emerged in response to specific issues and contexts and bring with them a diversity of interests, worldviews and strategies for change. In creating our network, our hope is to broaden our engagement with other actors advocating for change and strengthen our collective efforts. For academics, this requires moving further beyond the collective comfort zone of peer-reviewed publications, working with the media and those with political expertise, and learning from and supporting other stakeholders with a common vision.

## Ethical issues

 Not applicable.

## Competing interests

 Authors declare that they have no competing interests.

## Authors’ contributions

 MM led the writing of the original draft. All authors contributed equally to reviewing and editing the present manuscript.

## Funding

 This work was supported by funding from the Faculty of Health Sciences (FHS) at the American University of Beirut (AUB), as part of a grant funded by the International Development Research Centre (IDRC). MM is funded by the Irish Health Research Board [Grant Number ARPP-2020-002]. CGD is supported by a pre-doctoral contract for training in health research from the Carlos III Health Institute (FI18/00073).
